# Video review by utilizing asynchronous video communication platform

**DOI:** 10.1002/jgf2.70005

**Published:** 2025-02-21

**Authors:** Yuki Otsuka, Eiko Mitsuda, Yukichika Yamamoto, Atsushi Kato, Mano Soshi, Masaya Higuchi, Mikako Obika, Fumio Otsuka, Tadayuki Hashimoto

**Affiliations:** ^1^ Department of General Medicine Okayama University Graduate School of Medicine, Dentistry and Pharmaceutical Sciences Okayama Japan; ^2^ Kato & Namiki‐dori Hospital Okayama Japan; ^3^ BonBon, Inc Kyoto Japan; ^4^ Harvard Medical School Boston Massachusetts USA; ^5^ Department of Medicine, Division of Palliative Care and Geriatric Medicine Massachusetts General Hospital Boston Massachusetts USA; ^6^ Department of Emergency Medicine Brigham and Women's Hospital Boston Massachusetts USA; ^7^ Department of General Medicine Osaka Medical and Pharmaceutical University Osaka Japan

**Keywords:** feedback, general medicine, video review

## Abstract

**Background:**

Video review is widely recognized as an effective method for teaching communication; however, it can increase educators' workload and learners' stress.

**Methods:**

We utilized Tsucom, an online platform developed by BonBon, Inc., which enables asynchronous video communication instead of traditional styles. An 11‐min and 42‐s consultation video from a fifth‐year resident was uploaded, and 10 physicians provided 30 text‐based feedback.

**Results:**

In this pilot survey, the utility and ease of use were rated 4.4 and 4.1 out of 5, respectively.

**Conclusions:**

While asynchronous online video reviews provided flexibility and greater participation, challenges remain, and further trials and evaluations were deemed necessary.

## Graphical abstract

We utilized Tsucom, an online platform, which enables asynchronous video communication, for video review teaching. An 11‐min and 42‐s consultation video from a fifth‐year resident was uploaded, and 10 physicians provided 30 text‐based feedbacks. While asynchronous online video reviews provided flexibility and greater participation with highly rated utility and ease of use, challenges remain, and further trials and evaluations were deemed necessary.

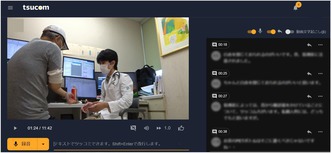

## 1 BACKGROUND

Feedback constitutes the foundation of an educational framework, playing a critical role in facilitating learning and improvement. Video review has emerged as an effective modality for delivering feedback. The practice of video review dates back to the 1960s and has been widely embraced over the years. This was even lauded as the gold standard for teaching communication.^1^ In fact, it has become an essential component of the general practice curriculum in many countries.^2^ The effectiveness of video review can be attributed to three factors. First, it allows evaluators to dedicate time for extensive observations. Second, practitioners are granted the opportunity to objectively view their own performance. Finally, it enables multidimensional assessment and provides affluent and comprehensive feedback.^3^

However, relying solely on self‐reviews without expert feedback is not effective. Expert feedback is vital to provide more nuanced and insightful evaluations. Despite these benefits, the implementation of video reviews incurs high costs, particularly for human resources. Educators are already experiencing workload challenges, and adding video reviews to their responsibilities exacerbates this issue.^4^ Moreover, video reviews can pose psychological stress for learners and raise concerns regarding patient privacy protection.^5^ The advent of asynchronous learning models offers potential solutions to these challenges. Asynchronous learning can offer a potential solution to allow both learners and educators to engage with the materials at their convenience. Particularly during the COVID‐19 pandemic, online education has gained attention, and the establishment of environments for asynchronous learning has been recommended.^6^ However, to date, no instances of video reviews being incorporated into asynchronous learning frameworks have been reported. Here, we report the first pilot study in which we conducted video reviews asynchronously and investigated the users' impressions and experiences.

## 2 METHODS

### 2.1 Development

**FIGURE 1:**
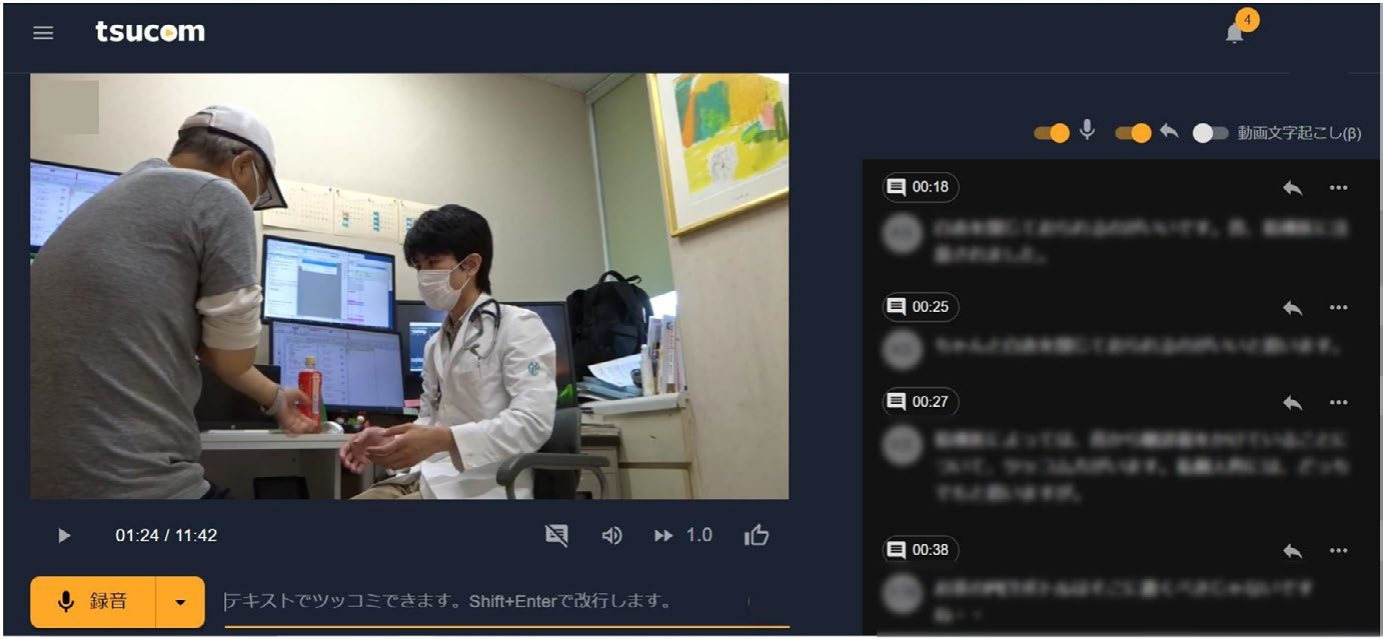
Utilization of Tsucom, an online platform for an asynchronous video review for outpatient clinic guidance of general medicine residents. A desktop image of Tsucom is shown. After obtaining patient consent, the consultation is filmed and the video file is uploaded to the platform, where it appears at the top of the screen. Physicians invited to the closed community can access the video at any time they like. Feedback can be provided below the video by using the orange button for voice input or the text box to the right for text input. Feedback provided by all participants is sorted chronologically and displayed on the right side, allowing it to be reviewed while watching the video (in the figure, personal information is obscured for privacy reasons).

We utilized Tsucom, an online platform developed by BonBon, Inc. (Kyoto, Japan), which supports asynchronous video communication.^7^ Tsucom allows members to be invited to a closed platform assigned to each group to access it independently. Videos can be uploaded to the platform, where members can watch them at their convenience and add reactions using text or voice. Users can also view and respond to comments from other members (Figure 1). In collaboration with the developers engaged in creating online platforms, we aimed to demonstrate the feasibility and potential benefits of incorporating video reviews into an asynchronous learning model, particularly highlighting its applicability and impact in medical education.

### 2.2 Implementation

An asynchronous video review was conducted to guide outpatient clinic work with a fifth‐year resident in a Japanese general medicine residency program, involving 10 supervising physicians. Some of these physicians are directly giving face‐to‐face supervision to the resident, while the others are those who have known the resident but do not meet with him on a routine basis. The video recorded a regular consultation with a patient in their 70s with chronic kidney disease, for whom the resident served as the primary physician. The video was recorded with the patient's consent, as is standard practice for video review. The 11‐min and 42‐s consultation video was uploaded to the online platform, Tsucom. The supervising physicians were asked for feedback online for more than 20 days by Google Form. Afterwards, the collected feedback and the consultation video were reviewed in a one‐hour reflection session via an online meeting.

### 2.3 Measurements

A web survey was conducted with physicians who provided feedback through asynchronous video reviews to collect their impressions and user experience with Tsucom. The utility of video review was rated using a Likert scale (1 = not useful at all and 5 = very useful). The ease of using Tsucom was similarly rated (1 = very difficult to use and 5 = very easy to use), and the responses were compiled as continuous variables. Additionally, they were asked to choose between face‐to‐face/synchronous and online/asynchronous video reviews, and to freely describe the advantages and disadvantages of asynchronous video reviews. The free‐text comments were aggregated as a descriptive study without any qualitative analysis.

### 2.4 Ethics statement

We obtained written consent for recording and publication from the patient and the resident. The survey data were compiled and analyzed with the approval of the Ethics Committee of Okayama University Hospital (No. 2401‐007), in accordance with the Declaration of Helsinki.

## 3 RESULTS

Among the 10 physicians, seven joined the asynchronous video review. There were 30 text feedback and no audio feedback. Responses to the survey were received from all physicians who provided feedback (response rate: 100%). They included three men and four women with a median postgraduate year of 11.

The mean score and standard deviation (SD) for Tsucom's utility in the video review were calculated as 4.4 and 0.79, respectively. Meanwhile, the mean score and SD for ease of use were 4.1 and 0.69, respectively. The advantages and disadvantages of asynchronous video review, which were freely described, are listed in Table 1. In response to the question of which was better: face‐to‐face/synchronous or online/asynchronous video review, all seven participants agreed that it was preferable to highlight the strengths of each approach.

**TABLE 1:** Advantages and disadvantages of video reviews gathered from supervising doctors via a web survey.

Advantages of asynchronous video reviews	Disadvantages of asynchronous video reviews
Each supervising physician can do it at their preferred time	It is hard to understand the patient's reactions and expressions
Multiple supervising physicians can react at the same time	Immediate feedback is not possible
The resident themselves can review the consultation video	It is likely that the freshness and immediacy of the review may decrease
One can reflect calmly afterwards, including candid reactions	There are concerns about security and risks of personal information leakage
The video can be watched repeatedly and paused to think	It is likely that individuals may respond with strong or harsh reactions
After reviewing other doctors' perspectives, additional feedback can be provided	

## 4 DISCUSSION

This study found that asynchronous video reviews were perceived as “useful” and “effective.” This assessment is critical in educational environments that are constantly seeking to integrate innovative teaching methods. In addition, the comments suggested that asynchronous video reviews could address some limitations of traditional methods and offer alternative instructional styles that are more conducive to certain learning environments or subject matter. This adaptability enhances their value as a complementary tool in an educator's repertoire.

Incidentally, the number of participants made this method the most remarkable. Owing to the COVID‐19 pandemic, only a few supervising physicians participated in the traditional video review at the site where this study was conducted. In our challenge, feedback was provided by seven supervising physicians, making it asynchronous. Additionally, the feedback may have resulted in new feedback that was not generated among the interaction between the supervising physicians or a calm environment. Although we still need face‐to‐face sessions, we believe that asynchronous online review in advance will shorten the time for face‐to‐face meetings and improve the efficiency of the discussion.

However, the study revealed limitations as well. Technological challenges, particularly the inability to capture patients' facial expressions, which are an essential part of communication, were noted. The possible solutions include adopting 360‐degree cameras or small action cameras that can capture both patient and physician, offering a more comprehensive view of clinical encounters. This advancement could considerably enhance the learning experience by offering a holistic view.

Legal challenges, predominantly concerning the security of patient information, were highlighted. As a safeguard, the developers confirmed that this system is compliant with Japanese ministry guidelines for handling patient information.^8^ However, because these guidelines are not tailored for educational purposes, the developer sought legal counsel to ensure there were no legal issues. Ensuring the use of secure cameras and stringent adherence to privacy guidelines remains critical. Nevertheless, continuous vigilance is necessary to address these legal and ethical considerations effectively.

Notably, this was a pilot study with a limited number of subjects. Therefore, future studies should be conducted on a larger scale, using more rigorous methodologies. This study suggests potential directions for future asynchronous video review. This includes conducting surveys on the current status of traditional video review programs, their implementation challenges, and their educational impact and burden on recipients. Identifying these issues could pave the way for asynchronous video reviews as an innovative solution that can potentially surpass traditional video reviews in terms of educational effectiveness. A comparative study could substantiate this finding by providing empirical evidence supporting the superiority of asynchronous methods over traditional video reviews. In addition, as a limitation of this study, the Resident who appeared in the video and the supervisor who evaluated it are included in the authors of this report; therefore, future evaluation is desired from a third‐party perspective.

Asynchronous video reviews present a promising avenue for the development of educational methodologies. Although this provides considerable advantages in terms of flexibility and instructional improvements, technical and legal challenges need to be addressed. Future research should focus on comparative studies to establish its efficacy over traditional methods and its continuous development to overcome current limitations.

## AUTHOR CONTRIBUTIONS

**Yuki Otsuka:** Data curation; investigation; writing – original draft preparation. **Eiko Mitsuda:** Data curation; resources. **Yukichika Yamamoto:** Investigation. **Atsushi Kato:** Investigation. **Mano Soshi:** Resources. **Masaya Higuchi:** Methodology. **Mikako Obika:** Supervision. **Fumio Otsuka:** Supervision. **Tadayuki Hashimoto:** Conceptualization; funding acquisition; project administration; supervision; review and editing.

## FUNDING INFORMATION

This work was supported by Japan Society for the Promotion of Science(JSPS) KAKENHI (Grants‐in‐Aid for Scientific Research) Grant Number JP22K21097.

## CONFLICT OF INTEREST STATEMENT

One of the authors (S.M.) is the chief executive officer and shareholder of BonBon Inc. The residency program that conducted the video review provided BonBon Inc. permission to use Tsucom free of charge.

## ETHICS STATEMENT

The survey data were compiled and analyzed with the approval of the Ethics Committee of Okayama University Hospital (No. 2401‐007) in accordance with the Declaration of Helsinki.
